# Facilitating fluency in adults who stutter

**DOI:** 10.1093/brain/awy075

**Published:** 2018-03-27

**Authors:** Jennifer T Crinion

**Affiliations:** Institute of Cognitive Neuroscience, University College London, UK

## Abstract

This scientific commentary refers to ‘Transcranial direct current stimulation over left inferior frontal cortex improves speech fluency in adults who stutter’, by Chesters *et al.* (doi:10.1093/brain/awy011).

This scientific commentary refers to ‘Transcranial direct current stimulation over left inferior frontal cortex improves speech fluency in adults who stutter’, by Chesters *et al.* (doi:10.1093/brain/awy011).

Being able to speak fluently is something most of us take for granted. However, for the estimated 70 million people worldwide with persistent developmental stuttering, speaking is a tense struggle to get words out. This can result in avoidance of speaking in some or many situations, with fear and anticipation of stammering affecting personal interactions, education and employment prospects (Boyle, 2015). As such, stuttering is not simply a speech difficulty but a serious communication problem. For children, behavioural interventions can work (Nye *et al.*, 2013). However, for the 1% of cases where stuttering persists to adulthood, changing the way speech is produced to maintain speech fluency is a particular challenge and there is a need for novel interventions (Howell, 2011). In this issue of *Brain*, Chesters and co-workers examine whether application of transcranial direct current stimulation (tDCS) concurrent with fluency training can improve speech fluency in people who stutter (Chesters *et al.*, 2018).

In a double-blind randomized controlled trial, 30 people who stutter underwent fluency training while receiving either anodal tDCS delivered over the left frontal cortices for 5 days (1 mA for 20 min/day), or sham stimulation. Outcomes were measured in terms of changes to stuttering severity both 1 and 6 weeks post-therapy. The behavioural intervention increased fluency immediately in all participants but only the people who stutter who received anodal tDCS maintained speech gains at both follow-up testing points. These results provide new insights into neuroplasticity in people who stutter in response to intervention, but at the same time raise a number of questions relating to (i) how to understand the behavioural consequences of tDCS; (ii) what role the left frontal cortices have in speech fluency; and (iii) how applicable these findings are to the goal of treating stuttering.

First, the speech changes observed by Chesters *et al.* took the form of reductions of disfluencies across two speaking tasks, reading and conversation 1 week post-therapy. In the reading task only, these reductions were maintained 6 weeks later. The authors interpret their results as evidence that speech samples taken during reading tasks provide a more sensitive measure of disfluency. This is certainly one potential account.

Yet we are still left with the question as to why the decreases in disfluency during the 5 days of intervention were greater during reading than conversation tasks only for the anodal tDCS group. From a methodological perspective, the behavioural invention was composed of two tasks: choral speech, which involved reading passages in unison with a live and then a recorded voice, and metronome-timed speech during cartoon narrations and conversations on random topics. Based on their intervention design, this means that Chesters *et al.* paired what one presumes is half their anodal tDCS dose with reading tasks that mirrored the reading outcome measures. It is not clear how long anodal tDCS was paired with the metronome conversation task. It has been suggested that anodal tDCS may induce facilitation when the task is well-trained or familiar, but such facilitation is not present during performance of a novel task (Dockery *et al.*, 2009). This may go some way towards explaining why Chesters *et al.* found a difference in outcomes between their two tasks.

TDCS itself cannot induce an over-threshold depolarization of neurons directly but can modulate the firing rate of the stimulated brain area. It will only induce the firing of neurons that are near threshold, which means that neurons not influenced by the task are less likely to discharge. In Chesters *et al.*’s well-practiced reading aloud task, the signal-to-noise ratio within the neural network is already above threshold. With anodal tDCS, the neural noise induced by stimulation is reduced so that the task input signal emerges clearly from the noise, thereby facilitating processing (Miniussi *et al.*, 2013). This is evocative of Hebbian-like plasticity mechanisms. The combination of anodal tDCS with reading fluency is similar to co-activation of a specific network modulating ongoing long-term potentiation—like changes that outlast the stimulation, leading to consolidation of changes in reading fluency performance. In their less-trained conversation task the context is different: the variability of the task likely meant variability of synaptic input function, meaning there was more background noise in the system and little consolidation of the neural networks. In this case, anodal tDCS would not help task performance as it would increase both the signal and the noise in the system, both being close to threshold. In this sense, anodal tDCS would not perturb the neural system supporting the conversation task’s behavioural processes nor lead to (long-term) conversation fluency change. In sum, tDCS requires ongoing learning in order to promote or modify plasticity to prime the behavioural system and produce corresponding specific effects in the cognitive system.

**Figure 1:**
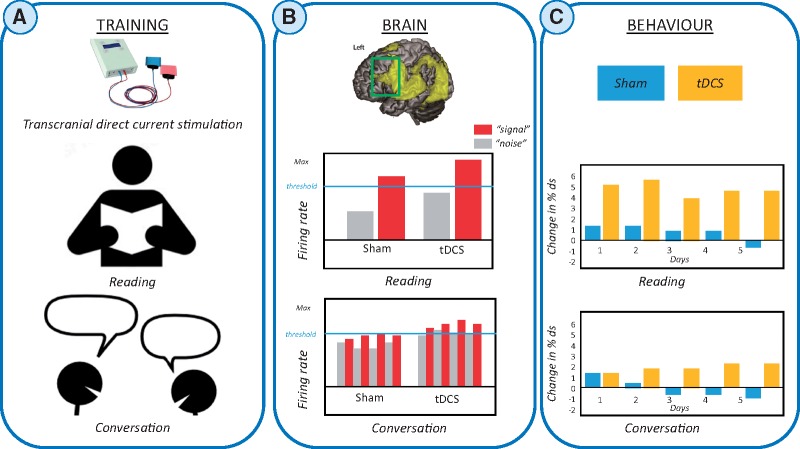
**Effects of behavioural interventions plus anodal or sham tDCS at brain and behavioural levels in persistent developmental stuttering.** (**A**) Training. *Top*: Photo of a standard tDCS kit that is paired with behavioural interventions. The speech fluency intervention used by Chesters *et al.* involved delivering tDCS (sham or anodal) concurrently with choral (reading based) and metronome-timed (including conversation) speech tasks. Speech fluency during reading and conversation tasks was then assessed after each day of intervention (*n* = 5) and at 1 and 6 weeks post-intervention. (**B**) Brain systems level. *Top:* Region-specific speech effects are illustrated relative to the location of the stimulating electrode. Yellow = left hemisphere BOLD response, overlaid on a canonical brain for the contrast ‘speaking relative to rest’ in a functional MRI study of healthy subjects. Green box indicates the approximate edge of the electrode (35 cm^2^) placed over the left frontal cortex. *Middle and bottom:* Illustrations of the possible neural response for reading and conversation tasks in the left frontal cortices underneath the stimulating electrode. Those neurons that respond according to the task-goal are displayed as target signal (red), all other sources of activity that are not associated with the final task-goal are defined as neuronal noise (grey). Plots show the interaction between target signal and noise when subjects read aloud (*top*), or engage in a conversation (*bottom*). The threshold represents the minimum signal intensity for neurons to contribute to the final speech task. The tDCS plots represent possible effects of anodal stimulation on the neurons that fire in response to the task demands. A pattern can be seen in the interaction between the task state and tDCS-induced activity. (**C**) Behavioural after-effects of anodal tDCS (yellow) versus sham (blue). The final behavioural outcome each day is likely dependent on the final neuronal patterns as shown in **B** schematic plots. *Middle:* Reading performance. *Bottom*: Conversation performance. The bars indicate the mean change in percentage of disfluencies (ds), where a high number indicates more fluent speech.

Second, what can we conclude about the role of left inferior frontal cortices in speech fluency in people who stutter? Chesters *et al.* proposed that this is a key brain region to support fluent speech on the basis of previous functional and structural imaging studies in people who stutter showing it to be structurally anomalous (Watkins *et al.*, 2008) or functionally underactivated (Budde *et al.*, 2014). They targeted this region using a tDCS montage with the reference electrode placed over the right supra-orbital cortex and the anode electrode (standard 7 × 5 cm size) placed over the left inferior frontal cortices also encompassing the ventral sensorimotor and premotor cortices. Thence extensive brain areas (not just inferior frontal cortices) were stimulated by Chesters *et al.* and the current flow between electrodes was widely distributed, potentially including subcortical structures.

Complex behaviours like speech production recruit large-scale bilateral neural systems. TDCS may, therefore, modulate task-related connectivity of regions distant to the stimulation site as well as task-related areas beneath the electrodes. This implies that the net behavioural effects evolving after stimulation are likely based on a remodelling of the whole task-engaged network; specifically, in the case of speech fluency, the behavioural effects reflect complex and potentially bilateral network interactions rather than changes in a single left frontal speech region. Indeed, Neef and colleagues’ combined functional MRI-diffusion tensor imaging data suggest that right fronto-temporal networks play a compensatory role as a fluency-enhancing mechanism in people who stutter (Neef *et al.*, 2018). While Lu and colleagues found increased functional activation in left ventral inferior frontal cortices and insula on a reading task after a 7-day behavioural intervention (also reading-based) for stuttering (Lu *et al.*, 2017). Their data combined with Chesters *et al.*’s reading data suggest that customizing tDCS to the task-induced neural activation during training is likely to increase specificity of effects. Nonetheless, the spatial resolution of tDCS is very low. Whether and how reduction in disfluencies induced by anodal tDCS placed over left frontal cortices paired with fluency interventions relates to the connection strength of co-activated (hypo and hyper) bilateral frontal regions to other brain areas remains an open question. In people who stutter, left inferior frontal cortices may be neither the only nor the optimal site for neuromodulation to affect speech fluency intervention outcomes.

At present, there are no universally-accepted methods or ‘gold standards’ for the treatment of people who stutter against which new or experimental interventions can be compared and no clear criteria to assess treatment efficacy. While clinically meaningful outcome measures have not been established, most therapists, clinicians, and researchers in the field would probably agree that a treatment should be considered effective if: (i) subjects show significant improvement in trained tasks compared to untrained tasks; (ii) these behavioural effects persist beyond the training period; and (iii) any improvement in fluency-based measures generalizes to real-world contexts. Chesters *et al.* have shown that anodal tDCS paired with a fluency intervention was safe and well-tolerated in a sham-controlled study in 30 people who stutter. The group who had anodal tDCS paired with their training significantly improved speech fluency compared to those who received the fluency intervention alone. The fluency gains were maintained for up to 6 weeks after therapy on reading-based tasks that were arguably trained more during the tDCS intervention. The results were not only statistically significant but the standardized effect sizes were large using Cohen’s *d*. Given the small sample size and the low dose of intervention (5 days), these results are promising. That the participants did not report a significant improvement in their functional speech skills is perhaps not surprising. This may be because the outcome measure used, OASES (Yaruss and Quesal, 2006), focuses on the psychosocial impact of stuttering and as such is ill-equipped to detect increased speech fluency, or because increased speech fluency during reading may have little applicability in daily life.

In the search for more effective and longer-lasting interventions, combining training and brain stimulation seems reasonable. The appeal of tDCS is its portable, inexpensive, safe and relatively simple set-up. The challenge for the treatment of people who stutter is to take a clinically effective stuttering intervention, understand its neural mechanism of action and, from these data, identify a candidate site for neuromodulation. Only then will tDCS have the potential—not as a complete therapy in itself but as an adjunct to effective behavioural interventions—to improve therapeutic outcomes.
